# Cannabidiol in Dietary Supplements: Characteristics, Routes of Administration, Bioavailability, and Research Challenges

**DOI:** 10.3390/molecules31132385

**Published:** 2026-07-07

**Authors:** Angelika Talar-Śpionek, Edyta Juszczuk-Kubiak, Marek Roszko

**Affiliations:** 1 Department of Food Safety and Chemical Analysis, Prof. Wacław Dąbrowski Institute of Agricultural and Food Biotechnology—State Research Institute, 36 Rakowiecka Street, 02-532 Warsaw, Poland; angelika.talar-spionek@ibprs.pl; 2 Department of Biotechnology, Prof. Wacław Dąbrowski Institute of Agricultural and Food Biotechnology—State Research Institute, 36 Rakowiecka Street, 02-532 Warsaw, Poland; edyta.juszczuk-kubiak@ibprs.pl

**Keywords:** cannabidiol (CBD), bioavailability, safety, dietary supplements, novel foods

## Abstract

Cannabidiol (CBD) has gained increasing attention as an ingredient in dietary supplements and functional foods due to its potential health-promoting properties. As a major non-psychoactive phytocannabinoid derived from *Cannabis sativa* L., CBD has been associated with anti-inflammatory, antioxidant, and neuroprotective effects. However, its application in nutraceutical products remains challenging because of its low and variable oral bioavailability, limited long-term safety data, and ongoing regulatory requirements. In the European Union (EU), CBD-containing products are classified as Novel Foods and require a comprehensive safety assessment before market authorisation. This review summarises current knowledge on CBD in dietary supplements, providing an overview of its chemical characteristics, routes of administration, and the key factors influencing its absorption and pharmacokinetics. It also reviews the main CBD formulations available in dietary supplements, including oils, capsules, gummies, and e-liquids, with particular emphasis on their characteristics relevant to CBD stability and bioavailability, as well as current regulatory frameworks. Finally, the major scientific and technological challenges associated with CBD-containing dietary supplements are highlighted.

## 1. Introduction

In recent years, cannabinoids, particularly cannabidiol (CBD), have gained significant scientific and commercial interest as ingredients of dietary supplements and functional foods [1,2,3,4]. To date, hundreds of plant-derived cannabinoids have been identified, with CBD being the most extensively studied and well-characterized compound. CBD is derived from *Cannabis sativa* L. hemp varieties characterized by low or negligible levels of tetrahydrocannabinol (THC) [5,6,7]. The absence of psychoactive effects, together with the broad spectrum of potential biological activities of CBD, including anti-inflammatory, antioxidant, and stress-response-modulating properties, has contributed to the rapid expansion of the market for CBD-containing products. In parallel, growing awareness of the relationship between diet, the endocannabinoid system, and overall health has further increased interest in CBD among both consumers and the scientific community [6,8]. The growing interest in cannabinoids is reflected in the dynamic expansion of the European industrial hemp market, which is projected to exceed USD 20 billion by 2033 [1]. However, the commercialisation of CBD-containing products is currently outpacing the accumulation of reliable scientific data on their safety, bioavailability, metabolism, and potential interactions with other bioactive compounds and medicinal products. In the EU, hemp-derived products, including cannabidiol, are classified as Novel Foods and therefore require a comprehensive risk assessment prior to market authorisation, including evaluations of safety, stability, and compositional consistency [6,9,10]. Despite the growing number of experimental and clinical studies, significant gaps remain in our knowledge of optimal forms of CBD supplementation, route-dependent bioavailability, metabolism, and long-term safety.

This review provides a systematic overview of the current knowledge on the use of CBD in dietary supplements, with particular emphasis on its chemical properties, available formulations, routes of administration, bioavailability, metabolism, safety, and the current regulatory framework governing CBD-containing dietary supplements.

## 2. Classification and General Characteristics of Phytocannabinoids

Cannabinoids constitute a diverse group of compounds that interact with the endocannabinoid system and can be classified by origin as phytocannabinoids, endocannabinoids, and synthetic cannabinoids [11,12,13]. Phytocannabinoids are naturally occurring terpenophenolic compounds produced by *C. sativa* L., whereas endocannabinoids are endogenous lipid mediators synthesised in humans and animals [11,14]. Synthetic cannabinoids are laboratory-derived molecules designed as agonists of the cannabinoid receptors [15].

More than 120 phytocannabinoids have been identified in *C. sativa* L., with cannabidiol (CBD), Δ9-tetrahydrocannabinol (THC), cannabigerol (CBG), and cannabichromene (CBC) being among the most extensively studied [16,17,18]. These compounds are synthesised and accumulated primarily in glandular trichomes located on the flowers and leaves of female plants. Phytocannabinoid biosynthesis begins with the formation of cannabigerolic acid (CBGA), the central precursor from which the major cannabinoid acids, including tetrahydrocannabinolic acid (THCA), cannabidiolic acid (CBDA), and cannabichromenic acid (CBCA), are generated through enzyme-catalysed reactions [19,20]. Subsequent decarboxylation during processing or storage yields the corresponding neutral cannabinoids, such as THC, CBD, and CBC [16,17,18]. The biological effects of phytocannabinoids are mediated through interactions with cannabinoid receptors and other molecular targets. CB1 receptors are predominantly expressed in the central nervous system and regulate neurotransmitter release, pain perception, mood, memory, and motor function [21,22]. In contrast, CB2 receptors are mainly found in immune cells and peripheral tissues, where they modulate inflammatory and immune responses [23,24]. In addition to CB1 and CB2, phytocannabinoids interact with several non-cannabinoid targets, including transient receptor potential vanilloid 1 (TRPV1) channels, G protein-coupled receptor 55 (GPR55), and peroxisome proliferator-activated receptor gamma (PPARγ). Through these pathways, phytocannabinoids influence a wide range of physiological processes, including inflammation, nociception, energy metabolism, cell proliferation, and maintenance of homeostasis [17,18,25].

## 3. Cannabidiol (CBD)

### 3.1. Chemical Structure and Physicochemical Properties of CBD

**Figure 1:**
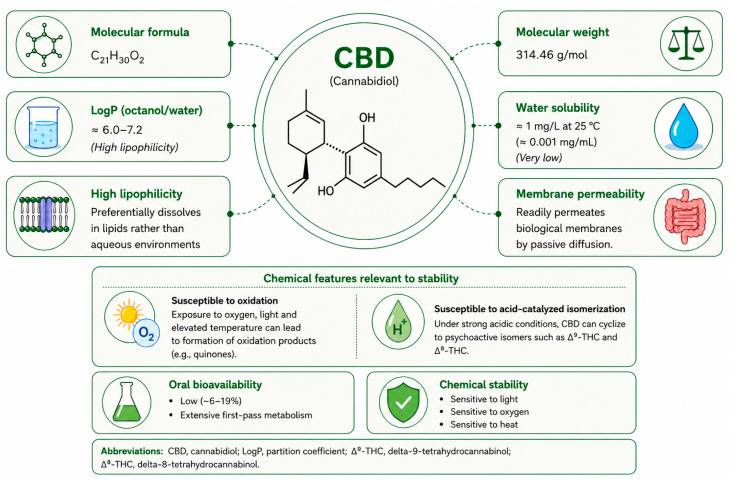
Chemical structure and key physicochemical properties of cannabidiol (CBD).

CBD is a primary non-psychoactive phytocannabinoid found in *C. sativa* L. [26]. It is an organic compound with the molecular formula C_21_H_30_O_2_ and a molecular weight of 314.46 g/mol, belonging to the terpenophenol class [27,28]. Its structure features an aromatic resorcinol ring linked to a terpene moiety of isoprenoid origin [29]. The presence of hydroxyl groups imparts phenolic character and influences chemical reactivity, while the hydrophobic alkyl chain increases lipophilicity (logP ~6–7.5), facilitating penetration of biological membranes [30,31]. Unlike THC, CBD lacks a closed pyran ring, a structural feature that contributes to its distinct pharmacological profile and absence of psychoactive effects [32]. CBD exhibits structural, geometric, and stereochemical isomerism due to the presence of chiral centres and conformational flexibility [33]. The naturally occurring form is (−)-trans-cannabidiol, characterised by a (1R,6R) absolute configuration within the terpene moiety [34,35,36]. This stereochemical arrangement influences receptor binding and interactions with biological targets, and even subtle changes in molecular conformation may alter the biological activity of CBD isomers [37,38] (Figure 1).

CBD is highly lipophilic and markedly hydrophobic, which strongly influences its physicochemical behaviour and biological performance [39,40] (Table 1). CBD is nearly insoluble in water (~0.001 mg/mL at 25 °C) but exhibits considerably higher solubility in lipids and vegetable oils. In medium-chain triglyceride (MCT) oil, CBD can reach concentrations of 90–100 mg/mL, and similarly high solubility has been reported in rapeseed and olive oils, making these carriers particularly suitable for lipid-based formulations and nanoemulsion systems [41,42]. CBD is also highly soluble in organic solvents such as ethanol (20–50 mg/mL, depending on solvent composition), dimethyl sulfoxide (DMSO; >100 mg/mL), propylene glycol (PG; approximately 58 mg/mL at 37 °C), and propan-2-ol (isopropanol), where solubility may reach 10–14% (*w*/*w*) under experimental conditions [43,44]. These physicochemical characteristics significantly influence formulation design, absorption, and bioavailability, making lipid-based delivery systems particularly suitable for CBD administration.

### 3.2. Chemical Stability of CBD

The chemical stability of CBD is an important consideration in the development of pharmaceutical formulations, dietary supplements, and cosmetic products [45,46]. CBD is susceptible to environmental factors such as acidic pH, oxygen, light exposure, and elevated temperatures, which may lead to degradation, isomerisation, and oxidation, potentially affecting its biological activity and safety profile.

#### 3.2.1. Isomerisation Under Acidic Conditions

Under acidic conditions, CBD can undergo cycloisomerisation to form psychoactive isomers such as Δ^9^-THC and Δ^8^-THC [34]. In laboratory settings, this process occurs in the presence of acids (e.g., HCl) at pH 1–5 and temperatures of 37–70 °C, during which the open-ring structure of CBD cyclises, yielding psychoactive THC isomers [47]. The extent of conversion depends on pH, temperature, and exposure duration, and can reach 70–90% under strongly acidic conditions [34,48].

A study by Merrick et al. [49] demonstrated that cannabidiol underwent substantial degradation in simulated gastric fluid (SGF) containing 1% sodium dodecyl sulphate (SDS). Approximately 85% of CBD degraded after 60 min of incubation, and more than 98% after 120 min. The primary degradation products were Δ^9^-THC and Δ^8^-THC, indicating that CBD may undergo chemical transformation into psychoactive cannabinoids under specific in vitro acidic conditions [49]. However, these findings were obtained using an in vitro experimental model and should not be directly extrapolated to in vivo conditions. The use of SDS, an anionic surfactant commonly employed to enhance the solubility of lipophilic compounds, may have influenced the extent of CBD degradation and the formation of transformation products. Therefore, the clinical relevance of these observations and their implications for central nervous system exposure remain uncertain [49,50].

Kinetic analysis showed that degradation followed first-order kinetics, with a rate constant of 0.031 min^−1^, confirming that the reaction rate was proportional to the current concentration of CBD in the reaction medium. This suggests that as the substrate concentration decreased, the degradation process slowed. The results suggest that under conditions simulating increased CBD solubility in the acidic gastric environment, significant conversion to psychoactive isomers may occur, thereby influencing the compound’s pharmacodynamic profile following oral administration [49,50]. Subsequent studies confirmed that the conversion of CBD to THC under simulated gastric conditions is strongly dependent on formulation and the presence of surfactants. In synthetic gastric fluid containing 1% SDS, a CBD nano-formulation was converted to Δ^9^-THC at a rate of 33–36%. However, under conditions more closely reflecting physiological settings, the conversion rate without SDS was substantially lower, reaching a maximum of 0.063% after 3 h of incubation. In the case of an oil formulation, conversion was practically undetectable (<0.0006%) [51]. These findings indicate that the extent of CBD conversion to THC in the gastrointestinal tract is limited and largely depends on CBD solubility and the presence of surfactants [51].

To improve the stability of CBD and minimise its degradation in the acidic gastric environment, modern formulation techniques have been developed, including self-emulsifying drug delivery systems (SEDDSs) and enteric coatings. SEDDSs enhance the solubility and bioavailability of CBD by forming fine emulsions in the gastrointestinal tract, thereby promoting small-intestinal absorption while limiting exposure to gastric acid [52,53,54]. Enteric coatings, in turn, remain stable at low gastric pH and dissolve at the higher intestinal pH, protecting the active compound from acid degradation and potential isomerisation into psychoactive compounds [49,54]. As a result, the stability of CBD after oral administration largely depends on the formulation used, highlighting the importance of appropriate formulation design to optimise its pharmacokinetic properties and safety profile.

#### 3.2.2. Oxidative Degradation of CBD

CBD is susceptible to oxidation, which represents one of the major pathways of its chemical degradation and may compromise the stability of CBD-containing products [45,55]. Exposure to oxygen, light, and elevated temperatures promotes the gradual conversion of CBD into CBD quinone and other oxidative degradation products that may possess biological activities distinct from those of the parent compound [30,56]. The oxidation process primarily involves the phenolic moiety of the molecule, resulting in the formation of quinone derivatives. Environmental factors, including temperature, pH, and solvent composition, can significantly influence the rate and extent of oxidation, thereby reducing the concentration of active CBD in pharmaceutical, food, and cosmetic formulations [30,45,56]. The formation of oxidative degradation products may affect both the safety and efficacy of CBD-containing products. Consequently, various strategies have been employed to minimise oxidative degradation, including antioxidant stabilisers, light-protective packaging, and controlled storage conditions [39,45,57]. Similar to acid-catalysed isomerisation, the susceptibility of CBD to oxidation is closely related to its molecular structure, particularly the presence of phenolic hydroxyl groups that facilitate redox reactions [34,58]. Therefore, understanding the mechanisms underlying oxidative degradation is essential for the rational design of stable CBD formulations and for maintaining product quality throughout storage and use.

### 3.3. Biological Properties and Molecular Targets of CBD

#### Interactions with the Endocannabinoid System and Other Molecular Targets

The biological effects of CBD are mediated through its complex modulation of the endocannabinoid system and its interactions with multiple receptors, ion channels, enzymes, and intracellular signalling pathways [59,60]. A key component of its mechanism of action involves modulation of cannabinoid receptor signalling, particularly through negative allosteric modulation of the CB1 receptor. In addition, CBD inhibits enzymes that degrade endocannabinoids, including fatty acid amide hydrolase (FAAH) and monoacylglycerol lipase (MAGL). In vitro studies have demonstrated that CBD inhibits FAAH (Ki ≈ 27.5 μM), leading to increased levels of anandamide (AEA) in the central nervous system, which has been associated with analgesic, anxiolytic, and neuroprotective effects without the psychoactive properties characteristic of THC [61,62]. Beyond the endocannabinoid system, CBD interacts with numerous molecular targets independently of CB1 and CB2 receptors. These include TRPV1, PPARγ, GPR55, and phosphodiesterase 9 (PDE9) [32,63]. CBD acts as an antagonist at GPR55 (Ki ≈ 0.7 μM), while PDE9 inhibition (IC50 ≈ 26 nM) increases intracellular cyclic guanosine monophosphate (cGMP) levels, which may contribute to neuroprotective and cognitive effects [63]. Activation of TRPV1 has been linked to modulation of nociceptive signalling and analgesic responses, whereas activation of PPARγ is associated with anti-inflammatory effects and regulation of immune-related gene expression [64,65]. At the cellular level, CBD also influences several signalling pathways involved in oxidative stress, inflammation, and cellular homeostasis [64]. It has been shown to modulate the activity of transcription factors such as nuclear factor kappa B (NF-κB) and to regulate the expression of genes associated with inflammatory and oxidative stress responses [66]. Furthermore, interactions with nuclear receptors, including PPARγ, contribute to the regulation of lipid metabolism, cell proliferation, and cellular differentiation [63,67]. Collectively, these diverse molecular interactions underlie the pleiotropic biological activity of CBD.

### 3.4. Metabolism and Drug–Drug Interaction of CBD

**Figure 2:**
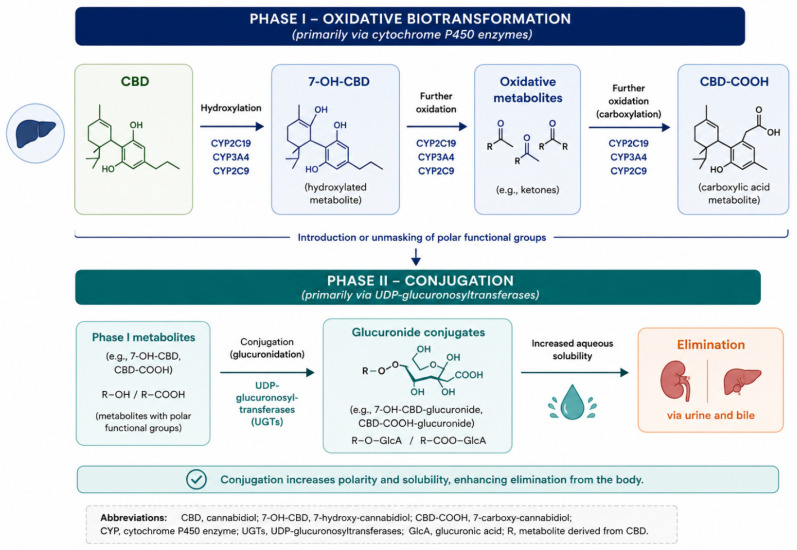
Phase I and phase II metabolism of cannabidiol (CBD) and major pathways involved in the formation and elimination of its metabolites.

The pharmacokinetic profile of CBD is strongly influenced by its extensive hepatic metabolism and its ability to modulate the activity of drug-metabolising enzymes. Following oral administration, CBD undergoes significant first-pass metabolism in the liver, contributing to its relatively low and variable systemic bioavailability [68]. The biotransformation of CBD occurs primarily through phase I oxidation reactions catalysed by cytochrome P450 (CYP450) enzymes, followed by phase II conjugation reactions that facilitate elimination from the body [57] (Figure 2).

Phase I metabolism is mediated predominantly by CYP3A4 and CYP2C19, with additional contributions from CYP2C9, CYP2D6, and CYP1A1/2. The primary metabolite formed during this process is 7-hydroxycannabidiol (7-OH-CBD), which retains biological activity and is considered an important contributor to the pharmacological effects of CBD. Further oxidation of 7-OH-CBD produces 7-carboxy-cannabidiol (7-COOH-CBD), the major circulating metabolite detected in plasma following oral administration [69]. Additional hydroxylated and oxidised metabolites have also been identified, although their pharmacological significance remains less well understood. Following phase I oxidation, CBD metabolites undergo phase II metabolism, primarily through glucuronidation mediated by uridine 5′-diphospho-glucuronosyltransferases (UGTs), including UGT1A9, UGT2B7, and UGT2B17 [70]. Conjugation with glucuronic acid increases the polarity and water solubility of CBD metabolites, facilitating their excretion through urine and bile. Consequently, both phase I and phase II metabolic pathways play crucial roles in determining CBD exposure, elimination, and overall pharmacokinetic behaviour [71].

In addition to serving as a substrate for CYP450 enzymes, CBD also inhibits several clinically important isoenzymes, particularly CYP3A4, CYP2C19, and CYP2C9 [72]. This inhibitory activity has significant implications for drug–drug interactions, as these enzymes metabolise a large proportion of commonly prescribed medications [73]. By reducing enzymatic activity, CBD may decrease drug clearance, increase plasma concentrations of co-administered medications, and increase the risk of adverse effects.

The most extensively documented interaction involves the antiepileptic drug clobazam [74,75]. CBD inhibits CYP2C19-mediated clobazam metabolism, increasing plasma concentrations of its active metabolite, N-desmethylclobazam. Clinical studies have shown that this interaction may lead to excessive sedation, somnolence, fatigue, and impaired psychomotor performance, often necessitating adjustment of the clobazam dose [76]. Elevated liver enzyme activities have also been reported during combined treatment with CBD and valproate, suggesting an increased risk of hepatotoxicity. However, this interaction appears to be predominantly pharmacodynamic rather than pharmacokinetic [77]. Clinically relevant interactions have also been observed with anticoagulants, particularly warfarin [78]. Since the more pharmacologically active S-enantiomer of warfarin is metabolised primarily by CYP2C9, inhibition of this enzyme by CBD may increase systemic exposure to warfarin and elevate the international normalised ratio (INR), thereby increasing the risk of bleeding complications [79]. Similar concerns apply to direct oral anticoagulants and antiplatelet agents that are metabolised via CYP-dependent pathways.

Another important group of interacting drugs includes immunosuppressive agents such as tacrolimus and cyclosporine [80,81]. Because CYP3A4 extensively metabolises these drugs and possesses a narrow therapeutic index, CBD-mediated inhibition of CYP3A4 may markedly increase their plasma concentrations. Clinical reports have demonstrated substantial increases in tacrolimus exposure during concomitant CBD administration, potentially leading to nephrotoxicity, neurotoxicity, hypertension, and other dose-related adverse effects [74,80]. CBD may also interact with benzodiazepines, antidepressants, antipsychotics, and other centrally acting medications metabolised by CYP3A4 or CYP2C19. Increased systemic exposure to these drugs may enhance sedation, dizziness, cognitive impairment, and psychomotor slowing [82]. Although the clinical significance of some of these interactions remains incompletely characterised, caution is warranted, particularly in patients receiving multiple medications or drugs with narrow therapeutic windows [79].

### 3.5. Biological and Therapeutic Effects of CBD

The interaction of CBD with multiple molecular targets results in a broad spectrum of biological and therapeutic effects. Numerous preclinical and clinical studies have demonstrated that CBD exhibits anticonvulsant, anti-inflammatory, analgesic, anxiolytic, antioxidant, and neuroprotective properties, making it a promising compound for various health-related applications [83,84]. The most well-established therapeutic application of CBD is in the treatment of drug-resistant epileptic syndromes [85]. Clinical studies have demonstrated its efficacy in reducing seizure frequency in patients with Dravet syndrome and Lennox–Gastaut syndrome, leading to the approval of CBD-based medicinal products for these indications in several countries [84].

Beyond epilepsy, CBD has attracted considerable interest as a potential therapeutic agent for neurodegenerative disorders [31,86]. Experimental studies suggest that CBD may help attenuate oxidative stress, neuroinflammation, and neuronal damage, which are key pathological processes involved in Alzheimer’s and Parkinson’s diseases [87]. Its antioxidant and neuroprotective properties have been proposed as potential mechanisms contributing to the preservation of neuronal function. CBD also exhibits significant anti-inflammatory activity by modulating immune responses and inflammatory signalling pathways. These effects have been investigated in the context of inflammatory bowel diseases, autoimmune disorders, and other chronic inflammatory conditions [63]. In addition, CBD may influence neurotransmitter systems involved in reward, motivation, and stress responses, indicating potential applications in the management of substance use disorders and addiction-related conditions [64]. Nonetheless, despite encouraging findings from preclinical studies and selected clinical trials, further well-designed human studies are required to establish optimal dosing regimens, long-term safety, and efficacy across different therapeutic indications. CBD also exhibits significant anti-inflammatory activity by modulating immune responses and inflammatory signalling pathways, including inhibition of NF-κB and MAPK-dependent cascades [64]. These effects have been associated with reduced levels of key pro-inflammatory cytokines, particularly tumour necrosis factor-α (TNF-α), interleukin-1β (IL-1β), and interleukin-6 (IL-6), as demonstrated in preclinical models of neuroinflammation and peripheral inflammation. In parallel, some experimental studies report increased levels of the anti-inflammatory cytokine interleukin-10 (IL-10), suggesting a shift toward a more anti-inflammatory immune profile [31]. These effects have been investigated in the context of inflammatory bowel diseases, autoimmune disorders, and other chronic inflammatory conditions [88,89]. In addition, CBD may influence neurotransmitter systems involved in reward, motivation, and stress responses, indicating potential applications in the management of substance use disorders and addiction-related conditions. Nonetheless, despite encouraging findings from preclinical studies and selected clinical trials, further well-designed human studies are required to establish optimal dosing regimens, long-term safety, and efficacy across different therapeutic indications [90].

## 4. CBD Formulations and Delivery Systems in Dietary Supplements

**Figure 3:**
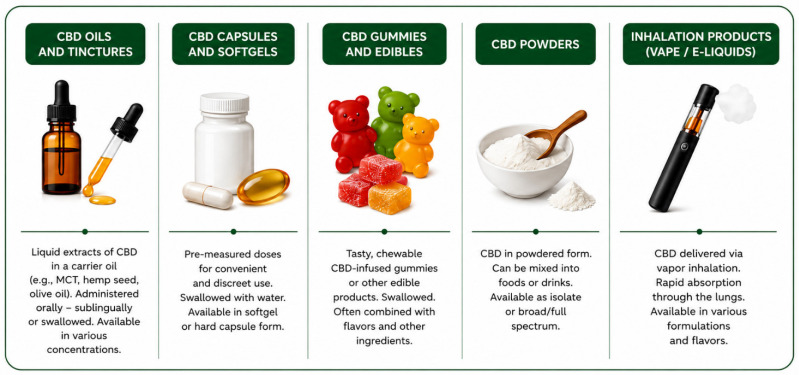
Common formulations of cannabidiol (CBD) used in dietary supplements.

CBD is commercially available in a wide range of dietary supplement formulations, including oils, capsules, softgels, gummies, powders, and inhalation products [57] (Figure 3). These formulations differ not only in their mode of administration and consumer convenience but also in their ability to influence CBD absorption and systemic exposure [91].

To overcome these limitations, considerable efforts have been directed toward developing advanced delivery systems to enhance CBD solubility, stability, and gastrointestinal absorption [42,92,93]. Recent studies have demonstrated that formulations such as nanoemulsions, SEDDSs, liposomes, and cyclodextrin inclusion complexes can substantially improve CBD bioavailability compared with conventional oral formulations [93,94]. In animal models, nanoemulsion-based systems increased the area under the concentration–time curve (AUC) severalfold, whereas SEDDSs increased the maximum plasma concentration (Cmax) by more than fourfold. Liposomal formulations may protect CBD from degradation within the gastrointestinal tract, while hydroxypropyl-β-cyclodextrin complexes improve both its aqueous solubility and chemical stability [95,96]. These findings highlight the critical role of formulation strategies in optimising CBD pharmacokinetics and, consequently, enhancing its biological and therapeutic potential. The following sections summarise the most common CBD supplement formulations currently available on the market and discuss their characteristics, advantages, and limitations.

### 4.1. CBD Oils

CBD oils are the most commonly used dietary supplements containing cannabidiol, owing to their relatively straightforward production process, precise dosing, and the high chemical stability of CBD in a lipid medium. These preparations are available in concentrations ranging from 5% to 30% (50–300 mg/mL) and are formulated with lipid carriers such as medium-chain triglycerides (MCTs) derived from coconut or olive oil, which serve as solvents and aid absorption [97]. The lipid nature of the carrier is vital because CBD is highly lipophilic (logP approx. 6–7), poorly soluble in water, and has a strong affinity for membrane structures, which facilitates its dissolution in the lipid phase while restricting its dispersion in the aqueous environment of the gastrointestinal tract [91]. Despite the use of lipid carriers, the oral bioavailability of CBD oils remains relatively low and variable, with estimates ranging from 6–20% [57].

An important factor influencing the pharmacokinetics of CBD oils is the route of administration. In clinical and supplementation practice, CBD oils are often administered sublingually, which allows partial bypass of first-pass metabolism by absorption through the highly vascularised oral mucosa. Sublingual administration provides faster attainment of the maximum plasma concentration (Cmax) and potentially higher bioavailability compared to conventional oral dosing. The extent of absorption depends on contact time with the mucosa, the administered dose, and the formulation characteristics. Pharmacokinetic studies indicate that part of the sublingually administered dose is inevitably swallowed and absorbed through the gastrointestinal tract, so total bioavailability is the sum of the fractions absorbed via the mucosa and the gut [98]. Both clinical and preclinical studies support the importance of formulation technology for CBD bioavailability. Sato et al. [93] demonstrated in healthy volunteers that liposomal CBD formulations significantly increased total systemic exposure (AUC) and Cmax compared with crystalline CBD. Cyclodextrin-based formulations also improved bioavailability, although to a lesser extent, mainly due to increased CBD solubility in the aqueous environment of the gastrointestinal tract and protection against degradation and presystemic metabolism. Similar findings were observed in preclinical studies. Nakano et al. [52] demonstrated in a rat model that CBD nano-emulsions with particle sizes below 200 nm and low polydispersity markedly improved pharmacokinetic parameters compared with traditional CBD oils. Nano-emulsions shortened the time to reach maximum plasma concentration (Tmax) from approximately 8 h to 2.4 h and increased total systemic exposure (AUC) by about 65%. Notably, nano-emulsion absorption was less dependent on bile, suggesting more efficient transport across the intestinal membrane, likely due to increased interfacial area and enhanced diffusion through the aqueous layer adjacent to enterocytes. Atsmon et al. [99] demonstrated in a clinical trial that formulations based on pro-nano-dispersion technology can substantially enhance CBD bioavailability in humans. Approximately 1.6-fold higher Cmax values and a shorter time to peak concentration were observed compared with traditional formulations. These results confirm that proper optimisation of physicochemical properties, including particle size, degree of dispersion, and lipid carrier characteristics, can significantly improve CBD pharmacokinetics [99].

### 4.2. CBD Capsules

Oral capsules, including gelatin (size 0/00) and hydroxypropyl methylcellulose (HPMC) vegetable capsules, are among the most common dosage forms used in CBD-containing dietary supplements. Their popularity results from convenient administration, accurate dosing (typically 10–100 mg CBD per capsule), protection of the active ingredient from light, oxygen, and moisture, and effective masking of the characteristic taste of CBD oils [57,97]. Despite these advantages, the oral bioavailability of CBD from conventional capsules remains limited because of its high lipophilicity, very low aqueous solubility, and extensive first-pass hepatic metabolism [100]. Pharmacokinetic studies in dogs have shown that CBD capsules formulated with MCT or olive oil can achieve maximum plasma concentrations (Cmax) of approximately 0.5–2 μg/mL, with Tmax values typically ranging from 3 to 5 h [32,101]. Considerable inter-individual variability has also been reported, reflecting differences in metabolic activity, physiological status, and dietary factors [100]. To improve oral absorption, advanced capsule formulations incorporating self-micro-emulsifying drug delivery systems (SMEDDS) and nanoemulsion technologies have been developed. These systems enhance CBD dispersion and maintain the compound in a solubilised state within the gastrointestinal tract, thereby improving its absorption [52,98]. Both preclinical and clinical studies indicate that such formulations can increase systemic exposure, resulting in higher Cmax and AUC values compared with conventional oil-filled capsules [52,99,102]. In addition to improved bioavailability, advanced capsule formulations offer greater dose standardisation and enhanced protection against oxidative degradation, making them promising delivery systems for CBD-containing dietary supplements and pharmaceutical products [32,97,102].

### 4.3. CBD Gummies

CBD gummies are a widely used oral dosage form of cannabidiol, valued for their high consumer acceptability, ease of administration, and ability to mask the inherently bitter taste of CBD effectively. Their palatable form and convenient dosing have contributed to their growing popularity among a broad range of consumers, including younger adults. These products typically contain gelatine as a gelling agent, together with sweeteners and bulking agents such as glucose–fructose syrup, maltitol, or sorbitol, and are formulated to deliver defined doses of CBD, most commonly ranging from a few to several tens of milligrams per unit [57,103]. Despite their favourable acceptability profile, conventional CBD gummies are associated with limited oral bioavailability, primarily due to the very low aqueous solubility of CBD, its high lipophilicity, and extensive first-pass hepatic metabolism. Within the gelatine-based matrix, CBD is often dispersed rather than fully dissolved, which may affect its physicochemical stability and dissolution behaviour under gastrointestinal conditions, thereby limiting absorption [99].

In a recent study, Johnson et al. [103] evaluated 56 commercially available CBD gummy products from the U.S. market using liquid chromatography–tandem mass spectrometry (LC–MS/MS). The results revealed that a substantial proportion of the products did not contain the labelled amount of CBD, while many also contained detectable levels of Δ^9^-THC. Considerable variability was observed both among different products and between individual units within the same package, indicating inconsistencies in dosing accuracy, product quality, and manufacturing standardisation [103]. To overcome the pharmacokinetic limitations of conventional gummy formulations, advanced delivery systems such as gelatine-based beadlets (matrix pellet systems) have been developed to improve CBD dispersion and physicochemical stability. These formulation approaches have been associated with enhanced pharmacokinetic performance, including increased maximum plasma concentration (Cmax) and area under the concentration–time curve (AUC), although the magnitude of the improvement varies with formulation design and study conditions [104].

### 4.4. Other CBD Products—Inhalation Forms

Inhalable forms constitute a significant yet less-regulated category of CBD-containing products, particularly so-called vape pens, which are mainly available online. Unlike medicinal products, these preparations show considerable compositional variability, with propylene glycol (PG) and vegetable glycerine (VG) being the most common carriers. PG acts as a solvent for cannabidiol, aiding its aerosolisation. However, experimental data suggest that PG/VG alone and their aerosol mixtures are not biologically inert; they may induce cytotoxicity and disrupt respiratory tract epithelial functions, including mucociliary clearance [105]. In vitro studies have also shown that aerosols generated from commercial CBD e-liquids can be more cytotoxic than preparations without cannabinoids and can provoke an inflammatory response in bronchial epithelial cells [106]. In turn, in vivo studies have demonstrated that inhaling CBD dissolved in PG can lead to inflammatory changes in the upper respiratory tract at high doses. Nevertheless, a relatively wide safety margin is observed at exposure levels typical for e-cigarette users [107].

In recent years, technological advances in CBD e-liquid formulation have become evident, including efforts to optimise PG/VG solvent systems to enhance cannabinoid stability, improve inhalation bioavailability, and minimise thermal degradation products [108]. Patent applications in this area suggest that the composition of the carrier phase, particularly the proportion of propylene glycol, is a key factor influencing the aerosol’s physicochemical properties and potential health risks. These risks are linked to changes in respiratory tract mucosal function and increased levels of carbonyl and hydroxyquinone compounds. Therefore, inhaled forms of CBD, particularly those available outside pharmaceutical regulation, remain an area of active research and significant debate regarding safety, due to the lack of quality standards, the instability of CBD during vapourisation, and the uncertain toxicological profiles of long-term inhalation of PG/VG combined with cannabinoids [109,110].

## 5. Safety Profile of CBD-Containing Foods and Dietary Supplements

### 5.1. General Safety and Adverse Effects of CBD

CBD used in dietary supplements and food products is generally well tolerated at typical consumer doses, usually 5–50 mg/day [111]. Clinical data indicate that the most common adverse effects are mild and include drowsiness, changes in appetite, diarrhoea, and gastrointestinal discomfort [57]. At higher oral doses (>100–200 mg/day), elevated liver enzymes (ALT, AST) have been reported, particularly with Epidiolex administered at 10–20 mg/kg body weight per day [112]. The EFSA report 2021 [113] recommends monitoring liver function in such cases and considering potential interactions with drugs metabolised by CYP2C9, CYP3A4, and CYP2C19; caution is advised in patients with liver disease [113]. The safety of oral CBD also depends on the formulation and bioavailability. Advanced formulations, such as nano-emulsions and SEDDS systems, increase systemic exposure by 2–4 times compared to traditional oils, potentially heightening the risk of adverse effects at equivalent nominal doses [93,104]. Moreover, CBD content in food products and supplements on the market shows significant variability, ranging from 30–150% of the declared dose. The presence of THC above 0.3%, heavy metals, or pesticides may further increase the risk of adverse effects [6,9,20,114,115]. Therefore, standardising dosage, implementing rigorous quality control measures, and ensuring product purity are critical for safeguarding consumer safety. Despite the generally good tolerability of CBD in dietary supplements, further long-term studies (>12 months) in healthy populations are necessary to assess its safety fully.

### 5.2. Safety of Alternative Routes of CBD Administration

Most currently available safety data for CBD are derived from studies investigating oral formulations, including dietary supplements and pharmaceutical preparations. In contrast, considerably less information is available regarding alternative routes of administration, particularly inhalation-based products. As the use of CBD-containing e-liquids and vaping products continues to increase, concerns have emerged regarding their toxicological profile and the lack of comprehensive long-term safety data. For inhaled products, such as e-cigarette liquids, additional risks arise from excipients (e.g., propylene glycol and vegetable glycerin), which may thermally degrade into compounds with known irritant and cytotoxic properties, including aldehydes and acrolein [105,110]. Furthermore, evidence indicates that CBD-containing aerosols may induce inflammatory responses in airway epithelial cells and exhibit greater cytotoxicity than the carrier substances alone [106]. The considerable variability in the composition of commercially available e-liquids, together with the lack of standardisation regarding product quality and stability, remains a significant concern [108]. Consequently, inhaled CBD formulations should be regarded as an area of increased toxicological uncertainty that warrants further investigation and rigorous quality control.

## 6. Legal Regulations on Dietary Supplements Containing CBD in the EU and Poland

### 6.1. Legal Aspects of CBD-Containing Food in the EU

In the EU, the legal status of CBD in foods and dietary supplements is governed primarily by Regulation (EU) 2015/2283 on novel foods [9]. Under this framework, only novel foods that have undergone a scientific safety assessment and have been authorised by the European Commission (EC) may be legally placed on the EU market. The Union list of authorised novel foods is established by Commission Implementing Regulation (EU) 2017/2470 and is updated as new authorisations are granted [114]. According to the EC’s Novel Food Catalogue, CBD and cannabinoid-containing extracts obtained from *C. sativa* L. are generally considered novel foods unless a documented history of significant consumption within the EU before 15 May 1997 can be demonstrated. The scientific and regulatory evaluation of CBD remains ongoing. In February 2026, EFSA updated its previous assessment and established a provisional safe intake level of 0.0275 mg/kg body weight per day, corresponding to approximately 2 mg/day for a 70 kg adult. However, this value applies only to highly purified CBD preparations (≥98% purity) that do not contain nanoparticles and whose manufacturing process and genotoxicity profile have been adequately characterised. EFSA also identified persistent uncertainties regarding the potential effects of CBD on the liver, endocrine system, nervous system, and reproductive health. In addition, the Authority concluded that the available evidence remains insufficient to establish safety for individuals younger than 25 years of age, pregnant and lactating women, and persons taking concomitant medications [116]. Although numerous applications for CBD-containing novel foods have been submitted to the EU, many remain under evaluation or have been withdrawn, suspended, or terminated due to insufficient safety data. According to the 2026 EFSA update, more than 200 applications for CBD as a Novel Food had been submitted to the EU by 27 August 2025, of which 17 were undergoing risk assessment by EFSA [116]. These ongoing evaluations reflect the continued scientific and regulatory uncertainty surrounding CBD and demonstrate that its legal status within the EU is still evolving. Future authorisation of CBD-containing foods will depend on the generation of robust toxicological, pharmacokinetic, and clinical data to address the safety concerns identified by EFSA. Consequently, the European regulatory approach remains precautionary and science-based, requiring a comprehensive demonstration of safety before market authorisation can be granted.

### 6.2. The Legal Status of CBD-Containing Foods in Poland

In Poland, the legal status of CBD-containing products is determined by the interaction between national narcotics legislation and EU food law. Under the Polish Act of 29 July 2005 on Counteracting Drug Addiction, fibre hemp is defined as *Cannabis sativa* L. containing no more than 0.3% total THC and THCA on a dry-weight basis [117]. Consequently, CBD obtained from compliant hemp varieties is generally not classified as a controlled substance. However, compliance with narcotics legislation does not automatically permit the use of CBD in foods or dietary supplements. Under EU food law, CBD and cannabinoid-containing extracts are classified as novel foods under Regulation (EU) 2015/2283. Therefore, any CBD-containing product intended for human consumption must undergo a safety assessment and obtain authorisation at the EU level before it can be legally marketed. In Poland, the absence of such authorisation effectively prevents the lawful marketing of CBD-containing dietary supplements under the Act of 25 August 2006 on Food Safety and Nutrition (Journal of Laws 2006 No. 171, item 1225, as amended).

Certain hemp-derived products with a documented history of consumption, including hemp seeds, hemp seed oil, hemp seed flour, and defatted hemp seeds, are not considered novel foods and may be marketed provided they comply with applicable safety requirements, including negligible levels of psychotropic cannabinoids. Nevertheless, CBD-containing products remain widely available on the Polish market, reflecting a regulatory grey area created by differences between national hemp legislation and the requirements of EU novel food law. In practice, supervisory authorities, including the State Sanitary Inspection, may undertake actions such as product withdrawal, market restrictions, or reclassification of products that do not comply with applicable legislation [115]. The future regulatory status of CBD-containing foods in Poland will largely depend on developments at the EU level. Positive EFSA opinions and future authorisations of CBD-containing novel foods may provide a clear legal pathway for market access, while simultaneously establishing harmonised safety and quality requirements. Compared with the EU, regulatory approaches adopted in other jurisdictions differ considerably. For example, the United Kingdom has implemented a national novel food authorisation process for CBD products, whereas Canada regulates CBD within a broader cannabis framework. These differences illustrate the dynamic nature of CBD regulation worldwide and highlight the need for harmonised, evidence-based regulatory frameworks that ensure both consumer safety and market transparency.

## 7. Future Perspectives and Research Challenges

Due to its very low aqueous solubility and variable oral bioavailability, improving CBD delivery remains a major focus of current research. Among the most promising approaches are advanced formulation technologies, including nanoemulsions, liposomes, SEDDSs, and cyclodextrin inclusion complexes. In particular, hydroxypropyl-β-cyclodextrin (HP-β-CD) has attracted considerable attention for its ability to enhance CBD solubility, stability, and dissolution in aqueous media, thereby potentially improving its bioavailability and therapeutic efficacy [94,96]. The continued development and optimisation of such delivery systems may contribute to more effective and predictable CBD supplementation.

Although CBD remains the most extensively studied phytocannabinoid, increasing attention is being directed toward other non-psychoactive cannabinoids. Preclinical studies suggest that compounds such as cannabigerol (CBG), cannabichromene (CBC), cannabinol (CBN), and tetrahydrocannabinolic acid (THCA) possess diverse biological activities, including anti-inflammatory, antibacterial, neuroprotective, and analgesic effects, as well as potential modulation of metabolic and tumour-related pathways [118]. However, the current evidence is derived predominantly from in vitro studies and animal models, and clinical data regarding their efficacy, safety, and pharmacokinetic properties in humans remain limited [118,119,120]. Among these compounds, CBG has emerged as a particularly promising candidate [118]. As a biosynthetic precursor of major phytocannabinoids, CBG interacts with multiple molecular targets, including cannabinoid-related pathways, transient receptor potential (TRP) channels, and serotonergic signalling systems, suggesting a broad spectrum of potential biological effects [121]. Similar to CBD, however, its practical application is limited by poor aqueous solubility and variable bioavailability, highlighting the need for advanced delivery systems and formulation strategies [119]. Consequently, well-designed clinical and pharmacokinetic studies are required to better characterise its therapeutic potential and safety profile.

Another emerging area of interest is the so-called “entourage effect”, which proposes that cannabinoids may interact with other constituents of *C. sativa* L., including terpenes and flavonoids, potentially influencing the pharmacological activity of cannabis-derived products [122]. Although this concept has attracted considerable attention, the underlying mechanisms and clinical relevance of such interactions remain insufficiently understood and warrant further investigation.

Future research should focus not only on improving cannabinoid bioavailability through innovative formulation approaches but also on generating high-quality clinical evidence regarding efficacy, long-term safety, pharmacokinetics, and potential interactions among cannabis-derived compounds. Addressing these challenges will be essential for supporting the safe and evidence-based use of cannabinoids in dietary supplements and other health-related applications.

## 8. Materials and Methods

### Search Strategy and Data Resources

This review was conducted as a structured narrative review of the literature on cannabinoids in dietary supplements, with particular emphasis on CBD. A narrative review approach was selected because the topic encompasses multiple disciplines, including cannabinoid chemistry, pharmacokinetics, formulation technologies, safety assessment, and regulatory frameworks. Furthermore, the available literature is highly heterogeneous in study design, products investigated, analytical methodologies, outcome measures, and source types. Consequently, a formal systematic review and quantitative synthesis were considered inappropriate for addressing the broad objectives of this review. The methodological approach was informed by published recommendations for conducting narrative literature reviews and evidence syntheses in health and life sciences [123].

A comprehensive literature search was conducted between October 2025 and May 2026, with the final search update performed in May 2026. Electronic databases, including PubMed, Scopus, and Web of Science, were systematically searched. To maximise coverage, additional searches were conducted using Google Scholar, ScienceDirect, PubMed Central (PMC), and the Directory of Open Access Journals (DOAJ). Citation tracking and manual screening of reference lists from relevant publications were also performed to identify additional studies not retrieved through database searches. In addition, official scientific opinions, guidance documents, and regulatory reports issued by the EFSA, the EU, and relevant Polish public authorities were reviewed to capture the current regulatory landscape of cannabinoid-containing products. The search primarily focused on publications from the previous decade, although earlier studies were included when considered foundational to the understanding of cannabinoid chemistry, metabolism, pharmacokinetics, toxicology, or regulatory classification. Search terms were combined using Boolean operators (AND, OR) and included the keywords “cannabidiol”, “CBD”, “phytocannabinoids”, “dietary supplements”, “food supplements”, “novel food”, “bioavailability”, “pharmacokinetics”, “nanoemulsion”, “self-emulsifying drug delivery systems”, “capsules”, “gummies”, “hemp extract”, “regulation”, “European Union”, and “Poland”. Example search strings included (“cannabidiol” OR “CBD”) AND (“dietary supplements” OR “food supplements”) and (“CBD”) AND (“bioavailability” OR “pharmacokinetics”).

Eligible sources included original research articles, clinical trials, pharmacokinetic studies, systematic and narrative reviews, scientific opinions, regulatory documents, and legal acts published in English or Polish. Conference abstracts without full-text availability, duplicate records, and publications not directly related to cannabinoid dietary supplements, bioavailability, safety, or regulatory issues were excluded. Commercial market reports were not used as primary evidence for scientific or regulatory conclusions, although they were consulted when relevant for contextual information. Retrieved publications were screened for relevance through title, abstract, and full-text assessment. Priority was given to peer-reviewed original studies, clinical investigations, systematic reviews, and official regulatory documents. When multiple publications addressed similar topics, preference was given to the most recent evidence, unless older studies represented seminal contributions to the field. Particular attention was paid to data concerning routes of administration, formulation-dependent bioavailability, metabolism, safety, and the regulatory status of CBD-containing products in the EU and Poland. Owing to the substantial heterogeneity of the available evidence, findings were synthesised narratively rather than quantitatively.

## 9. Conclusions

CBD has emerged as one of the most widely used cannabinoid ingredients in dietary supplements due to its potential anti-inflammatory, antioxidant, neuroprotective, and anxiolytic properties. However, its effectiveness is strongly influenced by its physicochemical characteristics, particularly its low aqueous solubility, limited oral bioavailability, and extensive first-pass metabolism. Consequently, formulation technologies play a critical role in determining the absorption and biological activity of CBD. Advanced delivery systems, including nanoemulsions, liposomes, SEDDSs, and cyclodextrin-based formulations, have demonstrated considerable potential to improve CBD stability, bioavailability, and therapeutic performance.

Despite the rapid expansion of the CBD supplement market, important scientific, regulatory, and safety challenges remain. Variability in raw material quality, extraction methods, cannabinoid composition, and product labelling may significantly affect both efficacy and safety. Furthermore, current regulatory frameworks remain fragmented, particularly regarding product standardisation, quality requirements, and long-term safety assessment. Although CBD is generally well tolerated, adverse effects and clinically relevant interactions with medications metabolised by cytochrome P450 enzymes have been reported, highlighting the need for careful use, especially among individuals receiving concomitant pharmacotherapy.

As CBD-containing supplements become increasingly accessible to consumers, greater emphasis should be placed on consumer awareness, transparent product labelling, and healthcare professional supervision. The widespread use of CBD products without medical guidance may increase the risk of inappropriate dosing, unrealistic health expectations, and potential interactions with prescription medications, particularly when product quality is uncertain. Future research should focus on the standardisation of CBD formulations, the development of evidence-based quality-control criteria, and the conduct of well-designed clinical studies evaluating long-term safety, pharmacokinetics, efficacy, and drug–drug interactions. Addressing these challenges will be essential for ensuring the safe, effective, and scientifically supported use of CBD-containing dietary supplements.

## Figures and Tables

**Table 1:** Solubility of CBD in selected solvents and lipid carriers.

Substance	CBD Solubility
Water	~0.001 mg/mL at 25 °C
MCT Oil	90–100 mg/mL
Rapeseed Oil/Olive Oil	90–100 mg/mL
Ethanol	20–50 mg/mL
Dimethyl Sulfoxide (DMSO)	>100 mg/mL
Propylene Glycol (PG)	58 mg/mL at 37 °C
Isopropanol (Propan-2-ol)	10–14% (*w*/*w*)

## Data Availability

No new data were created or analyzed in this study. Data sharing is not applicable to this article.
